# Asthma

**Published:** 1896-06-27

**Authors:** Patrick Watson Williams

**Affiliations:** Physician to Out-Patients and for Diseases of the Throat, Bristol Royal Infirmary


### Medical Progress and Hospital Clinics.

[*The Editor will be glad to receive offers of co-operation and contributions from members of the profession. All letters should be addressed to* The Editor, at the Office, 428, Strand, London, W.C.]

### ASTHMA.

By Patrick Watson Williams, M.D.Lond., Physician to Out-Patients and for Diseases of the Throat, Bristol Royal Infirmary.

(*Continued from page* 176.)

In 1892, when speaking (in connection with the subject of angina pectoris) on the clinical import of chronic arterial high tension, I submitted that the constant variations in arterial tension, the constant activity of the vaso-motor mechanism, which is presided over by the medullary vaso-motor centres, constitute one of the most striking instances of our physiological adaptability to our ever-varying environment. Is it pushing analogy too far to suggest that the constant activity of the broncho-motor mechanism, which is presided over by medullar centres, similarly constitute an instance of our physiological adaptability to our varying environment, using the term in its widest sense? If the analogy be admitted, we readily see how in predisposed and neurotic subjects the normal bronchial tension passes into bronchial spasm, and that conditions which tend to exhaust the nerve centres induce in them a hyper-æsthetic state, a want of tone in which there is an increased and abnormal response to slight stimuli. Just as an overcrowded stomach, excessive exercise, sudden emotion, or accumulation of uric acid in the blood may induce an attack of angina pectoris, while in the normal individual they only cause a physiological rise in arterial tension; so these and other like causes induce in the asthmatic a paroxysm, while in the normal individual they, perhaps, only cause healthy bronchial tension.

I forbear to push the analogy further, but I have referred to it because it bears so strongly on the therapeutical principles I believe to be essential for successful treatment of asthma. By successful treatment I do not mean the relief of the attacks, but the entire disappearance of the asthmatic tendency. Just as I have contended before that in angina pectoris or Bright's disease by the too frequent and uniform resort to remedies which act by dilating the arteries, we render futile any physiological effort to raise arterial tension, so do I now submit that it is most unscientific, and in principle radically wrong, to seek to cure asthma by means of drugs which undoubtedly relieve the spasm, but act by paralysing the muscular coats of the bronchioles and arterioles. For as long as the action of such drugs persists the physiological functions of the contractile bronchial tubes are completely abrogated, and when their transient effect passes off the broncho-motor mechanism is in a more unstable condition than before; that is to say, the patient who resorts to lobelia, belladonna, stramonium, or morphine and so forth for the relief of the spasms becomes only the more prone to subsequent attacks. What, indeed, can be more pernicious than the use of such anodynes by the asthmatics? Surely we know too well that these remedies are like opium to the opium smoker, and that while keeping the patient in a feeble and irritable condition they only increase the tendency to asthma and place anything like cure out of the question. Unfortunately, experience proves that there are many asthmatics who have so long developed the paroxysmal habit that palliative measures are our only resource, just as in well-developed or long-standing cases of angina pectoris we have no option but to keep the arterial tension low. Yet, if it is possible to do so, our aim should be to avoid all remedies which act as vaso- or broncho-dilators and adhere to tonic remedies and hygienic measures such as strychnine, phosphorus, arsenic, cold bathing, fresh air, and regular exercise, while removing all factors which tend to excite the attacks, such as indigestion, excess of uric acid in the blood, and any peripheral sources of reflex irritation of the implicated centres.

And now one word as to the influence of the nasal passages on asthma. There is a tendency, especially on the part of some rhinologists, to regard nasal disease as a prime and essential factor in the etiology of asthma, and, just because there are many facts and observations which lend weight to such a view, we must carefully avoid being too much "led by the nose" on this point.

One of the most ardent supporters of the nose origin theory of asthma is Bosworth. In forty-six cases of perennial asthma he found intra-nasal disease in all, and remarks: "I have never known a case of asthma to occur in other than obstructive lesion of the nose or upper air passages." This I can readily understand, for the simple reason that the nasal passages are part of the respiratory tract, and are comprised in the asthmatic region; consequently we must not be surprised that in twenty-six cases out of the forty-six thickening of the nasal mucous membrane was found, for it probably only corresponded to the alterations in the bronchial mucous membrane. In other words, the nasal disease, like the bronchial, is the result, I believe, rather than the cause of asthma. Equally do I believe that polypus of the nose associated with asthma is, at least, sometimes the result and not the prime cause of asthma. Space alone prevents my supporting this contention by clinical evidence, but I may mention that in thirteen cases of asthma seen this year, polypi, or a so-called myxomatous condition of the mucosa, was present in four. On the other hand, I see a larger number of patients with polypi who do *not* suffer from asthma.

It is, however, very remarkable what a large proportion of asthmatic paroxysms are cut short by spraying the nose with cocaine, even when there is no objective nasal disease present. There is then, undoubtedly, a close connection between the nose and asthma; possibly the bronchial tension is regulated by the influence of the air inspired on the nose passages. One thing I can positively assert from practical experience, viz., that stimulation of the nasal mucous membrane by a superficial cauterization or other means will altogether keep off or greatly modify the attacks of asthma in a large percentage of cases, and thus enable one to pursue a tonic treatment of asthmatics without resorting to the usual asthmatic sedative inhalations and remedies. I wish distinctly to say I do not advocate the intra-nasal treatment of all cases of asthma; each case must be treated on its merits. And if the galvano-cautery be used, it must be used with great care and moderation, otherwise the mucous membrane will be destroyed and the diseased condition may be aggravated instead of relieved. Its use should never be followed by any pain whatever.

